# Supplementary material for: MdWRKY120 Enhance Apple Susceptibility to Alternaria alternata
Source: Plants (Basel). 2022 Dec 5;11(23):3389. doi: 10.3390/plants11233389 (PMC10004717; doi:10.3390/plants11233389)
Supplement: Supplementary file 1 [file plants-11-03389-s001.zip › plants-2042355-supplementary.pdf]

Supplemental Table S1. Primers used for vector construction and qRT-PCR of *MdWRKY120.*

Primers name	Sequence （5’-3’）	Description
MdWRKY120-F	GTCGA*C*ATGGACTACTCAGCTGCATAT	Primers of cloning and overexpression vectors
MdWRKY120-R	GGTACCTCAGTAAGTATTGCGTTGAAG	
Sense*WRKY120*-F	TCTAG*A*TTGGATCTTAATTCCTTGCCT	Primes of RNAi vector forward fragments
Sense*WRKY120*-R	GTCGACTTGTTTGTGTTGTTGTTGCTG	
Anti*WRKY120*-F	GGTACCTTGTTTGTGTTGTTGTTGCTG	
Anti*WRKY120*-R	GAGCTCTTGGATCTTAATTCCTTGCCT	
MdWRKY120-GFP-F	GTCGACATGGACTACTCAGCTGCATAT	Primers of constructing GFP fusion vector
MdWRKY120-GFP-R	GGTACCGTAAGTATTGCGTTGAAG	
MdWRKY120-QRT-F	TTGACGGTGATAGGAGACA	qRT-PCR primers
MdWRKY120-QRT-R	TGAAATCTTAGCCTTGACG	qRT-PCR primers

Supplemental Table S2. Prediction of putative *cis*-regulatory elements of *MdWRKY120* promoter

cis-regulatory element	Position（strand）	Sequence	Function
5UTR Py-rich stretch	-369（+）	TTTCTTCTCT	*cis*-acting element conferring high transcription levels
A-box	-313（+）	CCGTCC	*cis*-acting regulatory element
ACE	-779（+）	ACGTGGA	*cis*-acting element involved in light responsiveness
TGAGTCA motif	-126（+）	ATACAAAT	associated to the TGAGTCA motif
Box 4	-708（-）	ATTAAT	part of a conserved DNA module involved in light responsiveness
Box I	-381（+）<br>-693（+）	TTTCAAA	light responsive element
Box-W1	-921（-）	TTGACC	fungal elicitor responsive element
CCGTCC-box	-313（+）	CCGTCC	*cis*-acting regulatory element related to meristem specific activation
CGTCA-motif	-1105（+）	CGTCA	*cis*-acting regulatory element involved in the MeJA-responsiveness
G-Box	-1113（+）	GACACGTAG T	*cis*-acting regulatory element involved in light responsiveness
GARE-motif	-872（+）	AAACAGA	gibberellin-responsive element
RY-element	-212	CATGCATG	*cis*-acting regulatory element involved in seed-specific regulation
Skn-1_motif	-903（-）<br>-1104（-）<br>-1036（-）	GTCAT	*cis*-acting regulatory element required for endosperm expression
TC-rich repeats	-782（-）	ATTTTCTTCA	*cis*-acting element involved in defense and stress responsiveness
TCA-element	-813（+）	CAGAAAAGG A	*cis*-acting element involved in salicylic acid responsiveness
TGACG-motif	-1105（+）	TGACG	*cis*-acting regulatory element involved in the MeJA-responsiveness
as-2-box	-668（-）	GATAatGATG	involved in shoot-specific expression and light responses
chs-CMA2a	-496（+）	TCACTTGA	part of a light responsive element
circadian	-81（-）	CAANNNNAT	*cis*-acting regulatory element involved

		C	in circadian control
W-box	-921（-）	TTGACC	
